# ^213^Bi-PAI2 conjugate selectively induces apoptosis in PC3 metastatic prostate cancer cell line and shows anti-cancer activity in a xenograft animal model

**DOI:** 10.1038/sj.bjc.6600179

**Published:** 2002-04-08

**Authors:** Y Li, S M A Rizvi, M Ranson, B J Allen

**Affiliations:** Center for Experimental Radiation Oncology, Cancer Care Center, St George Hospital, Gray St, Kogarah 2217, NSW, Australia; University of New South Wales, NSW 2052, Australia; Department of Biological Science, University of Wollongong, NSW 2522, Australia

**Keywords:** prostate cancer, PC3 cell line, targeted alpha therapy, α-particle emitter ^213^Bi, plasminogen activator inhibitor type 2, urokinase plasminogen activator

## Abstract

A novel α-particle emitting (^213^Bi) plasminogen activator inhibitor type 2 construct, which targets the membrane-bound urokinase plasminogen activator on prostate cancer cells, was prepared and evaluated *in vitro* and in a xenograft animal model. The PC3 prostate cancer cell line expresses urokinase plasminogen activator which binds to its receptor on the cell membrane; plasminogen activator inhibitor type 2 is bound to urokinase plasminogen activator/urokinase plasminogen activator receptor to form stable complexes. *In vitro*, the cytotoxicity of ^213^Bi-plasminogen activator inhibitor type 2 against prostate cancer cells was tested using the MTS assay and apoptosis was documented using terminal deoxynucleotidyl transferase-mediated deoxyuridinetriphosphate nick end-labelling (TUNEL) assay. *In vivo*, antiproliferative effects for tumours and prostate cancer lymph node metastasis were carried out in an athymic nude mouse model with a subcutaneous xenograft of PC3 cells. ^213^Bi-plasminogen activator inhibitor type 2 was specifically cytotoxic to PC3 cells in a concentration-dependent fashion, causing the cells to undergo apoptosis. A single local or i.p. injection of ^213^Bi-plasminogen activator inhibitor type 2 was able to completely regress the growth of tumours and lymph node metastases 2 days post subcutaneous inoculation, and obvious tumour regression was achieved in the therapy groups compared with control groups with ^213^Bi-plasminogen activator inhibitor type 2 when the tumours measured 30–40 mm^3^ and 85–100 mm^3^. All control animals and one of five (20%) mice treated with 3 mCi kg^−1^ ^213^Bi-plasminogen activator inhibitor type 2 developed metastases in the lymph nodes while no lymphatic spread of cancer was found in the 6 mCi kg^−1^ treated groups at 2 days and 2 weeks post-cell inoculation. These results demonstrate that this novel ^213^Bi-plasminogen activator inhibitor type 2 conjugate selectively targets prostate cancer *in vitro* and *in vivo*, and could be considered for further development for the therapy of prostate cancer, especially for the control of micro-metastases or in minimal residual disease.

*British Journal of Cancer* (2002) **86**, 1197–1203. DOI: 10.1038/sj/bjc/6600179 www.bjcancer.com

© 2002 Cancer Research UK

## 

Prostate cancer is the second most common cause of death from cancer in men after lung cancer. An estimated 180 400 new cases of prostate cancer were reported in the USA in 2000 with 31 900 deaths (Greenlee *et al*, 2000). Although surgical resection or radiotherapy is potentially curative for localised disease, advanced prostate cancer is associated with a poor prognosis. The major failure in the management of prostate cancer is the inability to inhibit the development of metastases that eventually result in death of the patient. In spite of conventional treatments, such as chemotherapy, radiotherapy and hormone therapy, the control of metastatic prostate cancer remains elusive. Thus, there is an urgent need for a therapy that can kill cancer cells in transit or at the pre-angiogenic stage.

The plasminogen activator system has been recognised to play an important role in tumour growth and metastasis (Pollanen *et al*, 1991; Andreasen *et al*, 1997; Schmitt *et al*, 2000). Bound to its specific cell surface receptor (uPAR), uPA efficiently converts the inactive zymogen plasminogen into the active serine protease, plasmin, which then cleaves either directly or indirectly extracellular matrix components including laminin, fibronectin and collagen (Pollanen *et al*, 1991; Schmitt *et al*, 2000). The activity of uPA is physically regulated by plasminogen activator inhibitors type 1 and 2 (PAI-1 and PAI-2) (Kruithof *et al*, 1995; Andreasen *et al*, 1997). PAI-2 is a member of the serine protease inhibitor (Serpin) superfamily and forms SDS-stable 1 : 1 complexes with uPA (Kruithof *et al*, 1995). It was suggested that cell surface-bound uPA is accessible to PAI-2, and PAI-2 can inhibit cancer cell invasion and metastasis (Kruithof *et al*, 1995; Hang *et al*, 1998).

α-particle therapy has been proposed for use in single-cell disorders such as leukaemias, lymphoma and micrometastatic carcinomas (Jurcic *et al*, 1997; McDevitt *et al*, 1998; Allen, 1999; Kennel *et al*, 1999), in which rapid targeting to cancer cells is possible. The radionuclide ^213^Bi (t_½_=46 min) emits an α-particle with short range (80 μm) and high linear energy transfer (LET) radiation which is about 100 times greater than that for beta particles (Allen and Blagojevic, 1997). This is manifested by a high relative biological effectiveness (RBE). The localisation characteristics of PAI2 allow ^213^Bi to deliver a large fraction of the total energy to the nucleus of the most malignant prostate cancer cells while irradiation of normal tissue around the target cell is greatly reduced or absent.

Using ^213^Bi-PAI2 conjugates, our group successfully targeted breast cancer *in vitro* and *in vivo* (Allen *et al*, 2000; Ranson *et al*, 2002). We show herein that ^213^Bi-PAI2 was specifically cytotoxic to PC3 cells in a concentration-dependent fashion, causing the cells to undergo apoptosis *in vitro*, whereas a therapeutic dose of ^213^Bi-PAI2 obviously regressed tumour growth and prevented metastases in the lymph nodes in animal models.

## MATERIALS AND METHODS

### PAI2

Human recombinant PAI2 (47 kD), reactive with the membrane-bound uPA, was provided by Biotech Australia Pty Ltd.

### Conjugation of bifunctional chelate to PAI2

The chelator, cyclic anhydride of diethylenetriaminepentacetic acid (cDTPA) was purchased from Aldrich Chemical Company. PAI2 and bovine serum albumin (BSA) were conjugated with cDTPA as described previously (Ranson *et al*, 2002).

### Generation of radioisotopes and ^213^Bi labelling of cDTPA-PAI2

The alpha particle emitting radionuclide, ^213^Bi, was produced from ^225^Ac/^213^Bi generator system. ^225^Ac column was purchased from the Department of Energy, USA. ^213^Bi was eluted from the ^225^Ac column with 250 μl of freshly prepared 0.15 M distilled and stabilised hydriodic acid followed by washing with 250 μl water. A time of 2–3 h is allowed for ^213^Bi to grow back in the generator for the next elution. Radiolabelling and purification of the PAI2 and BSA constructs with ^213^Bi were carried out using published methods (Rizvi *et al*, 2000; Ranson *et al*, 2002). The labelling efficiency of cDTPA-PAI2/BSA with ^213^Bi was up to 93%. Specific activity was in the range.

### Cell culture

The PC3 human prostate cancer cell line was originally purchased from American Type Culture Collection (Rockville, MD, USA), cultured in DMEM (Life Technologies, Inc., Grand Island, NY, USA) supplemented with 10% (v/v) heated-inactivated foetal bovine serum (FBS) and 1 : 100 penicillin/streptomycin. LN3 cells were originally obtained from Dr C Pettaway (MD Anderson Hospital) and maintained in 1 : 1 RPMI-1640 : F12-K (Life Technologies, Inc., Grand Island, NY, USA), supplemented with 10% (v/v) heated-inactivated foetal calf serum and 1 : 100 penicillin/streptomycin. Both cell lines were maintained in a humidified incubator at 37°C and 5% CO_2_. For all experimental procedures, sub-confluent cells that had been in culture for 48 h without a change of media were harvested by rinsing flasks twice with Dulbecco's phosphate buffered saline (DPBS) (pH 7.2) and then detaching with DPBS/0.5 mM EDTA at 37°C for 5 min. Cells were collected and resuspended in the appropriate buffer as described below.

### Monoclonal antibodies

Mouse anti-human uAP IgG antibody (No. 394) was purchased from American Diagnostica Inc (Greenwich, CT, USA). Rabbit anti-mouse IgG and alkaline phosphatase and anti-alkaline phosphatase (APAAP) complex were purchased from Dakopatts (Glostrup, Denmark). Mouse anti-human isotype control IgG1 monoclonal antibody and goat anti-mouse IgG fluorescein isothiocyanate (FITC)-conjugated monoclonal antibody were purchased from Silenus (Sydney, NSW, Australia).

### Flow cytometry

Indirect immunofluorescence staining was performed to detect cell-surface expression of uPA in the PC3 cell line as described previously (Ranson *et al*, 2002).

### *In vitro* cytotoxicity assay

The activities of ^213^Bi-PAI2 and ^213^Bi-BSA preparations were measured using a radioisotope calibrator and neutralised to pH 7.0 via the addition of 10% (v/v) 1 M NaHCO_3_ (pH 9.0). After this, six serial doses of ^213^Bi-PAI2 and one dose of ^213^Bi-BSA were immediately prepared in 100 μl DMEM/5% FBS and added to 96-well plates in triplicate containing 5×10^4^ PC3 cells (uPA^+^)/100 μl DMEM/5% FBS or 5×10^4^ LN3 cells (uPA^-^)/100 μl RPMI-1640/5% FBS. Controls were performed in triplicate in the same 96-well plate for each experiment and consisted of cDTPA-PAI2 and DMEM/5% FBS medium alone. The plates were then incubated overnight in a 5% CO_2_ atmosphere at 37°C.

The cells were then washed and incubated with 100 μl phenol-red free DMEM (without FBS) containing 20 μl of the CellTiter 96 Aqueous One Solution reagent. After 3 h incubation in a 5% CO_2_ atmosphere at 37°C, the reaction was stopped by the addition of 10% SDS, and the absorbency in each well was recorded at 490 nm using a SPECTRO max plate reader (BIO-RAD, Hercules, CA, USA). The absorbency reflects the number of surviving cells. Blanks were subtracted from all data and analysed using Prism software (GraphPad Software Inc, USA).

### TUNEL assay for apoptosis

The method was performed as described (Li *et al*, 2001). Briefly, the cultured PC3 cells were treated with ^213^Bi-PAI2 in different concentrations (1, 2.5, 5, 10, and 16 μCi) and with ^213^Bi-BSA, cDTPA-PAI2 or medium alone at 37°C overnight. After treatment, cells were washed with DPBS, harvested by centrifugation. Cytospin preparation were made with a Shando Cyto-centrifuge (Shando, Pittsburgh, PA, USA). Cell cytospins were prepared on glass slides using 3×10^4^ cells per 60 μl per slide. The cells were fixed in 4% paraformaldehyde at RT for 30 min. Apoptosis was measured using the TUNEL method (Gavrieli *et al*, 1992) with TdT-fragEL^tm^ *in situ* apoptotic detection kit according to the manufacturer's instruction (Oncogene Research Products, Boston, MA, USA). Specificity of TUNEL reactivity was confirmed by undertaking in parallel appropriate negative (omitting TdT from the labelling mix) and positive (treated HL-60 slides) control. The labelled cells were examined using a Leca light microscope (Leica microscope, Nussloch, Germany) at ×40 magnification. The results were expressed as a percentage of total cells staining positive for apoptosis.

### Animals and PC3 cell inoculation

Male 6–8 weeks old athymic nude mice, BALB/c (nu/nu), were obtained from Animal Resources Centre (ARC), Western Australia. The mice were housed and maintained in laminar flow cabinets under specific pathogen-free conditions in facilities approved by University of New South Wales (UNSW) Animal Care and Ethics Committee (ACEC) and in accordance with their regulations and standards. The ethical guidelines that were followed meet the standards required by the UK Coordinating Committee on Cancer Research Guidelines (Workman *et al*, 1998).

To establish s.c. tumours, 1.5×10^6^ PC3 cells were resuspended in 200 μl of DMEM serum-free medium and injected via 18-gauge needle into the s.c. space of the right shoulder region. Tumour progression was documented once weekly by measurements using calipers, and tumour volumes were calculated by the following formula: length×width×height×0.52 in millimeters (Gleave *et al*, 1991). Mice were euthanised by cervical dislocation while under ethrane anaesthesis.

### Treatment protocols

#### Experiment 1

^213^Bi-PAI2 at 2 days after PC3 cell inoculation. Four groups of five mice each received a single local injection of ^213^Bi-PAI2 at 25 or 50 μCi, ^213^Bi-BSA at 50 μCi (non-specific controls), or PBS buffer (controls). After 8 weeks, mice were sacrificed under anaesthesia, tumours, regional and distant lymph nodes were excised for histology.

#### Experiment 2

^213^Bi-PAI2 at 2 days after PC3 cell inoculation. Four groups of five mice each received single i.p. injections of ^213^Bi-PAI2 at 1.5, 3.0 and 6.0 mCi kg^−1^ or ^213^Bi-BSA at 6.0 mCi kg^−1^. After 8 weeks, the experiment was terminated.

#### Experiment 3

^213^Bi-PAI2 at 2 weeks post PC3 cell inoculation when tumour volumes were approximately 30–40 mm^3^. Three groups of five mice each received single i.p. injections of ^213^Bi-PAI2 at 3.0 and 6.0 mCi kg^−1^ or PAI2 (0.4 mg kg^−1^). After 6 weeks, the mice were euthanised.

#### Experiment 4

^213^Bi-PAI2 at 4 weeks post PC3 cell inoculation when tumour volumes were approximately 85–100 mm^3^. Groups of five mice each received single i.p. injections of ^213^Bi-PAI2 at 3.0 and 6.0 mCi kg^−1^ or cDTPA-PAI2 conjugate. After 4 weeks, mice were euthanised.

Tumour xenograft, lymph node and other organs (liver, spleen, kidney and lung) from sacrificed mice were immediately fixed in 10% neutral buffered formalin for paraffin section for uPA expression and H&E staining.

### Immunohistochemistry

The alkaline phosphatase anti-alkaline phosphate (APAAP) method was used to detect uPA expression in PC3 cells. Cell cytospins were made on glass slides as described above. Paraffin-embedded tissues were cut at 5 μm sections, mounted on gelatin-coated glass slides (Davis Gelatine, Australia) and then incubated for 20 min at 60°C. The slides were deparaffinised in xylene, followed by a graded series of alcohols (100, 95, and 75%) and rehydrated in Tris-buffer saline (TBS, pH 7.5). The following steps were also performed for the PC3 cell glass slides after a 20-min fixation in 4% paraformaldehyde at RT. The primary uPA (20 μg ml^−1^) was incubated for 2 h at RT or overnight at 4°C. After washing with Tris buffered saline (TBS), slides including tissue sections or cells were incubated with rabbit anti-mouse IgG (1 : 100 dilution) and APAAP complex (1 : 100 dilution) for 1 h and then stained by the addition of a fresh prepared alkaline phosphatase substrate included 0.2 mg ml^−1^ of naphthol AS-MX phosphate containing 0.1 mg ml^−1^ Fast Red TR and Levamisole in 0.1 M Tris HCl (pH 8.2) for 10 min. The positive cells appear pink.

### Statistical analysis

All numerical data were expressed as the average of the values obtained, and the standard deviation (s.d.) was calculated. For the *in vivo* studies, student's *t*-test was used to compare data and results with a *P* value less than 0.05 were considered statistically significant.

## RESULTS

### Expression of uPA in PC3 cells, PC3 xenograft tumour and lymph node metastases

**Figure 1:**
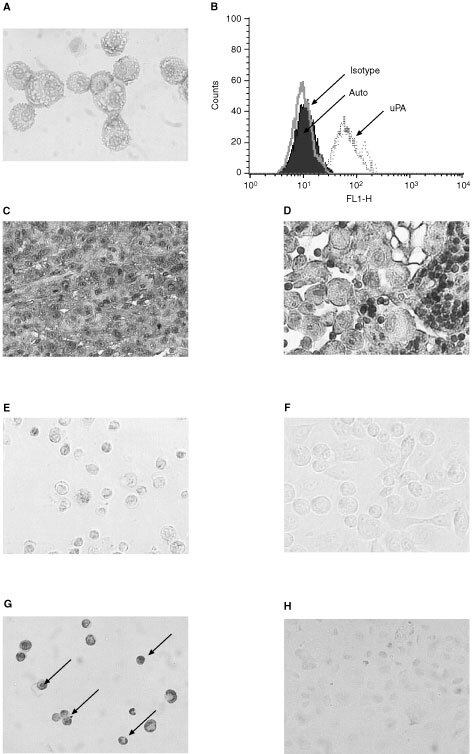
uPA expression in PC3 cells, PC3 xenografts and metastatic lymph nodes and TUNEL assay of PC3 cells *in vitro*. (**A**) PC3 cells are strongly positive to membrane-bound uPA. (**B**) Flow cytometry histogram of PC3 cells showing indirect immunofluorescence labelling of cell surface uPA. Auto: autofluorescence level; Isotype: mouse anti-human subclasses IgG1 control antibody; uPA: mouse anti-human uPA IgG1 antibody. (**C**) PC3 tumour xenograft cancer cells are strongly positive to uPA. (**D**) Cancer cells from metastatic lymph nodes are positive to uPA while lymphocytes are negative to uPA (small cells). The sections were from different mice. PC3 cells were either treated with ^213^Bi-PAI 2 (**E**) or untreated (**F**). After 36 h, the treated and untreated cells were fixed and processed for TUNEL assay. The cells were then visualised by light microscope. The arrows represent typical apoptotic cells with condensed or fragmented nuclei (**G**), while control cells show normal shapes (**H**). **A**, **C**, **E**, **F**, **G** and **H**: Magnification ∼×200. **D**: Magnification ∼×400.

Some 95% of PC3 cells stained positive by immunohistochemistry for uPA (Figure 1A

) while isotype control staining was negative (data not shown). We confirmed our immunostaining result with flow cytometry (Figure 1B). The results showed that the uPA histogram has a strong shift to the right compared with the negative and isotype controls, suggesting that uPA is expressed on the cell surface and provides a good target for ^213^Bi-PAI2 conjugate *in vitro*. In addition, we also investigated the expression of uPA in tumour xenografts, lymph nodes containing metastases and other organs (liver, spleen, kidney and lung) by immunohistochemistry. The staining intensity of cancer cells in tumour xenograft and metastasis lymph node was strong (Figure 1C,D); in contrast, the staining intensity of cells in other organs was very weak or negligible (data not shown).

### ^213^Bi-PAI2 is highly cytotoxic towards the PC3 cells *in vitro*

**Figure 2:**
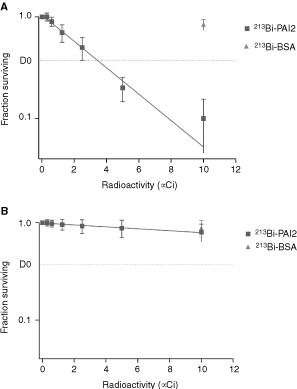
Representative cytotoxicity study of (**A**) PC3 and (**B**) LN3 prostate cancer cells. Fifty thousand cells were seeded in 0.3 ml medium. Cells were treated with varying concentrations of ^213^Bi-PAI2 or non-specific ^213^Bi-BSA, incubated overnight and cell survival measured by MTS assay at 24 h and expressed as a percentage of cell survival of control cells. Results are expressed as a mean per cent±s.d. of control plates containing non-specific α conjugate. Each point represents a mean of three experiments with each experimental point having triplicate wells.

^213^Bi-PAI2 was found to be highly toxic to PC3 cells in an activity concentration dependent fashion, whereas ^213^Bi-BSA showed only slight toxicity compared with ^213^Bi-PAI2 at the maximum activity used (Figure 2A

). No significant toxicity was observed with either cDTPA-PAI2 or PAI2 (data not shown). The D_0_ (37% cell survival) values with ^213^Bi-PAI2 was calculated to be 3.4 μCi. At the maximum dose of ^213^Bi-PAI2 (10 μCi) cell survival was reduced to 10% for PC3 cells while at the same dose of ^213^Bi-BSA cell survival was 92%. Additional experiments were designed to test ^213^Bi-PAI2 against a uPA-negative cancer cell line, LN3. The results indicated that the D_0_ value with ^213^Bi-PAI2 was 43.8 μCi, and a dose of 7.5 μCi ^213^Bi-PAI2 could kill no more than 15% of LN3 cells (Figure 2B). These results indicated that the alpha cytotoxicity was specific to PC3 cells.

### ^213^Bi-PAI2 induces PC3 cells apoptosis

PC3 cells were incubated with increasing concentrations of ^213^Bi-PAI2 (1∼10 μCi for 24 h) or controls. After treatment, ^213^Bi-PAI2-treated cells showed typical apoptotic morphology (Figure 1E), i.e., cells displayed shrinkage, became rounded and detached, whereas controls and non-specific ^213^Bi-BSA-treated cells did not exhibit apoptotic morphology (Figure 1F). Using an incubation of 5.0 μCi activity ^213^Bi-PAI2, 90.2±8.4% (mean±s.d., *n*=3) of the PC3 cells were TUNEL-positive, in comparision with 8.6±3.5% (mean±s.d., *n*=3) of the cells receiving ^213^Bi-BSA; 4.2±2.2% (mean±s.d., *n*=3) of the cells receiving cDTPA-PAI2; 2.1±0.5% (mean±s.d., *n*=3) of no treatment control. The free 3′ ends generated by apoptotic DNA cleavage were detected by TUNEL assay, in which the non-apoptotic cells stained green while apoptotic cells stained brown. The results were shown in Figure 1G,H.

### Tumour growth inhibition by ^213^Bi-PAI2

#### Experiment 1

**Figure 3:**
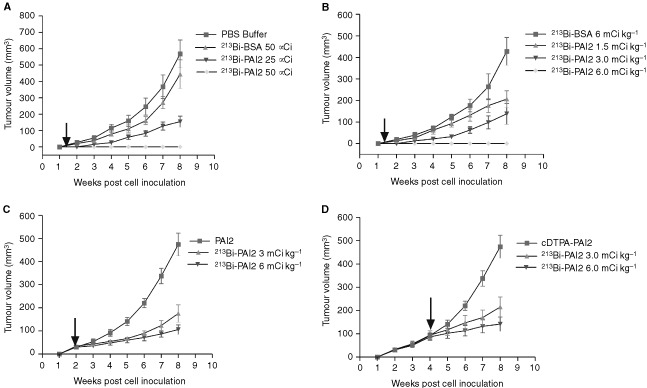
The effect of a single dose of ^213^Bi-PAI2 on the growth of s.c. xenografts of PC3 human hormone-independent prostate cancer in nude mice with local and systemic injections. The arrows indicate the start of each treatment. (**A**) Single intra-lesional injections of 50 and 25 μCi of ^213^Bi-PAI2 or 50 μCi of ^213^Bi-BSA non specific control and PBS buffer were given 2-days post inoculation. (**B**) Single i.p. injections of a series 1.5, 3.0, 6.0 mCi kg^−1^ of ^213^Bi-PAI2 or 6.0 mCi kg^−1^ of ^213^Bi-BSA were given 2-days post inoculation. (**C**) Single i.p. injections of 3 and 6 mCi kg^−1^ of ^213^Bi-PAI-2 or single equal amount of PAI2 (0.4 mg kg^−1^) were given when tumour volume was 30–40 mm^3^. (**D**) Single i.p. injections of 3 and 6 mCi kg^−1^ ^213^Bi-PAI2 or single equal amount of cDTPA-PAI2 were given when tumour volume was 85–100 mm^3^. No death related to toxicity occurred. Data are expressed as the mean±s.d. for five tumours.

We evaluated the antitumour activity of the ^213^Bi-PAI2 given as a single intra-lesional injection of 2 days post-inoculation at 25 and 50 μCi per animal with equal doses of non-specific ^213^Bi-BSA or the same volume of PBS buffer. Eight weeks after the administration of the ^213^Bi-PAI2, the cancer cells in the 50 μCi group were completely killed whereas the volume of PC3 tumours (154±33 mm^3^) in the 25 μCi group was significantly smaller than that PBS control group (568±85 mm^3^), corresponding to a 73% inhibition (*P*<0.01; Figure 3A

). No obvious inhibition of tumour growth was observed in mice that received ^213^Bi-BSA.

#### Experiment 2

A single i.p. injection of ^213^Bi-PAI2 was administered to mice at 2 days post inoculation at increasing doses of 1.5, 3.0 and 6.0 mCi kg^−1^. Eight weeks after treatment, PC3 tumour growth was completely inhibited at a dose of 6.0 mCi kg^−1^. The growth of tumours in the 3.0 mCi kg^−1^ group was delayed 1 week compared with that in non-specific control group (Figure 3B). The volumes for the 1.5 mCi kg^−1^ group (206±39 mm^3^) and in 3.0 mCi kg^−1^ group (138±48 mm^3^) were significantly smaller than that those in the non-specific control group (428±64 mm^3^) corresponding to 52 and 68% inhibition (*P*<0.01). During the treatment, no obvious toxicity was observed.

#### Experiment 3

Treatment was initiated when s.c. tumours had grown to a volume of 30–40 mm^3^. According to the results obtained from experiment 2, we selected doses of ^213^Bi-PAI2 at 3.0 and 6.0 mCi kg^−1^ for i.p. injection. As shown in Figure 3C, 6 weeks after therapy, ^213^Bi-PAI2 significantly inhibited the growth of PC3 tumours, as evidenced by tumour volumes of 106±20 mm^3^ in 6.0 mCi kg^−1^-treated group and 175±38 mm^3^ in 3.0 mCi kg^−1^-treated group as compared with controls (PAI2 alone) that measured 474±50 mm^3^ (78 and 63% reductions, *P*<0.001).

#### Experiment 4

Treatment was started when s.c. tumours had grown to a volume approximately twice as large as those in experiment 3. We used a similar treatment protocol as for experiment 3. As shown in Figure 3D, 4 weeks post ^213^Bi-PAI2, ^213^Bi-PAI2 significantly regressed the growth of PC3 tumours, as evidenced by tumour volumes of 148±31 mm^3^ in 6.0 mCi kg^−1^-treated group and 215±43 mm^3^ in 3 mCi kg^−1^-treated group as compared with controls (cDTPA-PAI2) that measured 474±50 mm^3^ (70 and 55% reductions, *P*<0.01).

### Effect of ^213^Bi-PAI2 on the development of lymph node metastases

Eight weeks after s.c. cell inoculation, mice in different groups were sacrificed and examined for lymph node metastases. As assessed by H&E staining under light microscopy, all control animals including PBS buffer groups and ^213^Bi-BSA non-specific groups developed metastases in the regional and distant lymph nodes, whereas the incidence of lymphatic spread was 60% (three out of five) for the 1.5 mCi kg^−1^ i.p. group 2 days post-cell inoculation; 40% (two out of five) for 25 μCi local 2 days post-cell inoculation and 3.0 mCi kg^−1^ i.p. post-tumour appearance as third week post-therapy groups; 20% (one out of five) in 3.0 mCi kg^−1^ i.p. 2 days post-cell inoculation and 6.0 mCi kg^−1^ i.p. post-tumour appearance third week groups. No metastases were found in the groups given local ^213^Bi-PAI2 2 days post-inoculation at 50 μCi, i.p. injection 2 days post-inoculation or post-tumour appearance first week at 6.0 mCi kg^−1^. The results are shown in Table 1

**Table 1:**
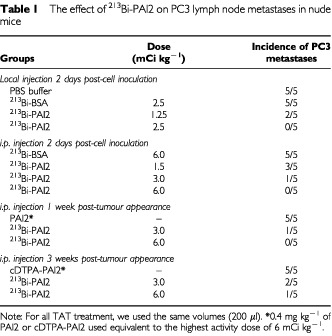
The effect of ^213^Bi-PAI2 on PC3 lymph node metastases in nude mice

.

## DISCUSSION

The present system relies on the targeting of ^213^Bi to uPA/uPAR tumour cells. As a result, a much greater fraction of the total energy is deposited in cells with alpha and very few nuclear hits are required to kill a cell. The expression of uPA/uPAR was found on a high percentage of human PC3 prostate cancer line (Crowley *et al*, 1993; Festuccia *et al*, 1998). Using immunocytochemistry and flow cytometry, we confirmed that uPA/uPAR was expressed on the surface of viable PC3 cells. In the present study, we also showed that PC3 tumours and their metastases highly express uPA, which had not been reported previously. Our findings indicate that the PC3 xenograft is an appropriate model for investigating the efficacy of alpha-PAI2.

PC3 cancer cells are a metastatic cell line while LN3 cells are non-metastatic and negative to uPA (Evans *et al*, 1997). PC3 cell kill *in vitro* proved to be both specific and activity dependent. Compared with untreated and non-specific controls, ^213^Bi-PAI2 exhibits high levels of antigen-selective cytotoxicity, requiring very few ^213^Bi atoms per target cell to kill PC3 cells. The D_0_ value of 3.4 μCi was found for 50 000 cells in 0.3 ml. Thus, 3.4 μCi or 12 000 Bq gives two decays per cell and kills 63% of the incubated cells. This indicates the specificity of the conjugate for cells expressing uPA. Additional data demonstrated that ^213^Bi-PAI2 did not specifically kill the uPA-negative LN3 prostate cancer cell line, supporting the fact that the ^213^Bi-PAI2 conjugate does not target or destroy tissue that does not express uPA. Therefore, it is clear that ^213^Bi-PAI2 is an effective and specific radiolabelled agent for ablation of individual PC3 prostate cancer cell *in vitro* whereas non-targeted cells are spared from the radiotoxicity arising from the alpha radiation.

The predominant mechanism by which radiation kills mammalian cells is the reproductive (or clonogenic) death pathway. DNA is the target, and double-stranded breaks in the DNA are regarded as the specific lesions that initiate this lethal response (Radford, 1986; Ward, 1994). Because of very high linear energy transfer (100 keV μm^−1^) of α-particles, cells have a limited ability to repair the damage to DNA (Nikula *et al*, 1999). Macklis *et al* (1992) reported that α-particles may kill cells by apoptotic mechanisms. In the present study, we demonstrated that ^213^Bi-PAI2 can induce high numbers of TUNEL positive cancer cells compared with control cells. The results suggest the lethal pathway of PC3 *in vitro* after α-PAI2 treatment is predominantly through apoptosis.

The four therapeutic experiments with s.c. xenografts carried out *in vivo* were designed to evaluate the antitumour activity and anti-metastatic effect of ^213^Bi-PAI2 after administration at different stages of tumour growth and to optimise the dosage regimen. Our findings indicate that a single local injection of ^213^Bi-PAI2 at a dose of 50 μCi (2.5 mCi kg^−1^) or a single i.p. injection of ^213^Bi-PAI2 at a dose of 6 mCi kg^−1^ can completely suppress tumour growth and prevent lymph node metastases for at least 8 weeks, while all control animals grew tumours and developed lymph node metastases. The growth regression of tumours and metastases was dose-dependent. This means that a proper dose of ^213^Bi-PAI2 can kill tumour cells through local or systemic TAT. A possible explanation of this finding could be that arresting the growth of primary tumours with ^213^Bi-PAI2, also prevents the dissemination of cancer cells to lymph nodes. At 2 days post inoculation, the seeded PC3 cells are not vascularised and not encapsulated, and isolated cells and preangiogenic lesions may be present.

According to our findings in experiments 3 and 4, a single systemic (i.p.) injection of ^213^Bi-PAI2 at doses of 3 and 6 mCi kg^−1^ during the growth of tumours, when the tumours were 35 mm^3^ (early stage) and 90 mm^3^ (late stage) in size, strongly regressed tumour progression for at least 5–7 weeks. Tumour volumes in the ^213^Bi-PAI2-treated groups decreased by 78 and 70% compared to the controls. These results suggest that ^213^Bi-PAI2 can still regress the growth of small, solid PC3 tumours but cannot eradicate them completely. This may be because the tumour size affects the efficacy of ^213^Bi-PAI2. It was reported that radioimmunoconjugate uptake usually decreases due to increased interstitial pressure (resulting from the lack of functioning lymphatic vessels) and restricted blood supply (Jain, 1991). The elevated interstitial fluid pressure may also act as a physiological barrier to the delivery of ^213^Bi-PAI2 conjugates by preventing extravasation of PAI2 as well.

One interesting finding of the experiments was that the elimination of lymph node metastases was dose-dependent. Another encouraging observation was that using the same amount of ^213^Bi-PAI2, targeting small primary tumours in the early stage can more effectively prevent lymph node metastases than larger tumours as a later stage. A possible interpretation of these findings could be that after absorption through the animal peritoneal vascular system, an effective dose of ^213^Bi-PAI2 can prevent the dissemination of the cancer and completely target and kill metastatic cancer cells or micrometastases that express uPA, leading to their complete regression. Prostate cancer cells expressing the prostate specific membrane antigen (PSMA) can be targeted by the J591 monoclonal antibody. McDevitt *et al* (2000) reported that ^213^Bi-J591 can regress the growth of LNCaP spheroids comprising ∼1000 cancer cells *in vitro*. This *in vitro* result also supports our *in vivo* targeting results. All of these findings indicate that ^213^Bi-PAI2 may target cancer cells in the very early micrometastatic stage, i.e. cells in transit or at the pre-angiogenic stage, while larger solid tumours exhibited a growth reduction, but not complete regression. Therefore, small multiple metastatic sites or minimum residual disease should be considered to be the most suitable targets for ^213^Bi-PAI2.

## CONCLUSION

We have shown that TAT using the ^213^Bi-PAI2 conjugate can: (a) kill PC3 prostate cancer cells in a dose dependent manner, requiring approximately at two decays per cell for 37% survival; (b) completely regress tumour growth at 2 days post inoculation using 2.5 mCi kg^−1^ for local TAT or 6 mCi kg^−1^ for systemic TAT; (c) delay tumour growth in a dose dependent way; (d) reduce lymph node metastases.
